# Extracellular vesicles including exosomes in cross kingdom regulation: a viewpoint from plant-fungal interactions

**DOI:** 10.3389/fpls.2015.00766

**Published:** 2015-09-23

**Authors:** Monisha Samuel, Mark Bleackley, Marilyn Anderson, Suresh Mathivanan

**Affiliations:** ^1^Department of Physiology, Anatomy and Microbiology, School of Life Sciences, La Trobe UniversityMelbourne, VIC, Australia; ^2^Department of Biochemistry and Genetics, La Trobe Institute for Molecular Science, La Trobe UniversityMelbourne, VIC, Australia

**Keywords:** exosomes, plant-fungal interaction, extracellular vesicles, cross kingdom regulation, secretome

Throughout evolution, plants and pathogenic fungi have been in a constant battle where fungi have developed new mechanisms to infect plants while plants have co-evolved to combat the infection. The early stages of plant-pathogen interactions occur in the intercellular spaces of the plant tissue and thus involve a myriad of secreted factors. Traditionally, all proteins released into the extracellular space were thought to be transported via the ER-Golgi dependent classical secretory pathway. However, non-classical secretion of proteins/RNA through extracellular vesicles (EVs) has recently been reported to contribute to the milieu of extracellular molecules that mediate plant-fungal interactions (Rodrigues et al., 2007; Meyer et al., 2009). EVs can be broadly classified into exosomes and ectosomes (Keerthikumar et al., 2015). Exosomes are secreted microvesicles (30–150 nm in diameter) of endocytic origin that are released by multiple cell types and are conserved across various species (Lotvall et al., 2014; Gangoda et al., 2015). In contrast, ectosomes or shedding microvesicles are larger (100–1000 nm in diameter) and bud off directly from the plasma membrane (Keerthikumar et al., 2015). For clarity, we will collectively refer to both types of membranous vesicles as EVs in this article.

Recent studies on mammalian systems have highlighted the role of EVs in cell-cell communication and the intercellular transport of cargo (proteins, nucleic acids, and carbohydrates) (Batista et al., 2011; Cossetti et al., 2014). Whilst the role of EVs in plant-fungal interactions is still poorly defined, this non-canonical secretory pathway has been proposed as an alternative route for the secretion of virulence and defense molecules by fungi and plants, respectively (Robatzek, 2007; Rodrigues et al., 2011). The basic requirement for successful host colonization is the establishment of a parasitic relationship between the fungal pathogen and the host. This requires the induction of specific defense mechanisms in the fungus for protection against the plant innate immune system (Hayes et al., 2013). Evasion or suppression of the plant defense response is thought to be regulated by virulence factors that are secreted from the fungus and act at the plasma membrane or in the cytoplasm of the plant cell (Rodrigues et al., 2008a). Interestingly, recent studies allude to the EV-mediated transport of virulence factors from the fungus into the host cell as a more efficacious delivery mechanism than simple diffusion (Rodrigues et al., 2008a; Silverman and Reiner, 2011). Similarly, in plants, when the integrity of the cell wall is threatened by a fungal pathogen, a response is mediated, at least in part, by multivesicular bodies (MVBs) (An et al., 2006b). In mammalian cells, it is well documented that fusion of MVBs with the plasma membrane results in the secretion of exosomes (Boukouris and Mathivanan, 2015; Gangoda et al., 2015). Though the production of MVBs may not always result in the secretion of EVs, the observation that plants produce MVBs in response to a fungal infection leads to the speculation that EVs may play a critical role in plant-fungal interactions. Here, we will discuss the current knowledge on EVs in the context of human-fungal interactions and their potential roles in plant-fungal interactions.

## Role of EVs in human-fungal interactions

Fungal EVs were first isolated from the human fungal pathogen *Cryptococcus neoformans* (Rodrigues et al., 2007). These EVs contained well known virulence factors such as the capsular polysaccharide glucuronoxylomannan (GXM) and the virulence regulator, glucosylceramide (Rodrigues et al., 2008a). Rodrigues and colleagues also reported the presence of several other pathogenicity-associated components that are delivered into the host via EVs. Furthermore, the isolated EVs were biologically active as they could invigorate phagocytes in the host and enhance their antimicrobial activity (Oliveira et al., 2010a). Other mammalian fungal pathogens including *Histoplasma capsulatum, Candida parapsilosis, Sporothrix schenckii*, and *Candida albicans* also deliver a variety of effector molecules in a similar manner (Albuquerque et al., 2008; Vargas et al., 2015; Gil-Bona et al., 2015b). Interestingly, the serum from patients with *H. capsulatum* infections contains antibodies to proteins that are present in the EVs produced by the pathogen indicating involvement of EVs in the host-pathogen interaction. Moreover, characterization of EVs from the human pathogens *C. neoformans, H. capsulatum* and *Malassezia sympodialis* has implicated them in the modulation of the host immune system and regulation of the host-pathogen interaction in favor of the fungus (Rodrigues et al., 2008b; Gehrmann et al., 2011).

## Role of EVs in plant-fungal interactions

A major component of plant-fungal interactions is the secretion of small proteins by both organisms. Plants produce pathogenesis related (PR) proteins, many of which inhibit fungal growth or directly kill fungal cells (Sels et al., 2008). Fungi secrete virulence factors encoded by the avirulence (AVR) genes (Stergiopoulos and De Wit, 2009; Rodrigues et al., 2014; Gil-Bona et al., 2015a). However, in spite of decades of research, it is still unclear as to how these proteins cross the plasma membranes and cell walls of both species. The AVR genes AVR_a10_ and AVR_k1_of the fungal pathogen *Blumeria graminis f. sp. hordei* encode proteins that lack signal peptides. Despite the lack of classical secretion signal, they still enter the cells in a susceptible host plant and are required for the pathogenicity of the fungus (Ridout et al., 2006). In mammalian systems, it is well established that certain proteins with and without signal peptides are transported via EVs (Kalra et al., 2012; Simpson et al., 2012). Hence, EVs could potentially mediate the transfer of these fungal virulence factors into plant hosts (Rodrigues et al., 2008b). However, further studies are required to understand this highly complex phenomenon.

The ability of a plant to mount a rapid defense response against potential pathogens is vital to its survival. Intercellular organelle rearrangements and structural modulation of the cytoskeleton with increased focal secretion of compounds lead to the formation of a physical barrier at the attack site that might prevent successful infection (Frey and Robatzek, 2009). These modifications may involve rapid and targeted delivery of molecules via EVs. Fungal infection enhances the formation of both intracellular MVBs and paramural vesicles between the plasma membrane and cell wall in the plant cells indicating the critical role of this secretory pathway in plant innate immune response (An et al., 2006a; Wang et al., 2014). For example, vesicular structures were associated with the accumulation of phenolic compounds and H_2_O_2_ that prevented pathogenic establishment of the powdery mildew fungus *B. graminis* in barley (*Hordeum vulgare*) leaves. Though the detection of plant MVBs at the site of infection provides indirect evidence of their role in plant defense (An et al., 2006a), more work is needed to define their molecular composition and whether they do indeed transport innate immunity proteins to the site of infection and/or into the fungal cell.

MVBs were first reported in the appressoria and haustoria of the powdery mildew fungus *B. graminis* (Hippe, 1985; Hippe-Sanwald et al., 1992). More recently, microscopic examination confirmed the presence of membrane bound vesicles at the biotrophic interface between *B. graminis* and the host plant (Micali et al., 2011). Furthermore, the haustorial complexes produced by *Golovinomyces orontii* in infected Arabidopsis leaves have vesicles and MVBs in the haustorial body (cytoplasm), paramural space, and extrahaustorial matrix. In addition, vesicle budding and fusion of MVB-like structures with the fungal plasma membrane has been observed in this interaction, although the microscopic evidence did not reveal whether the vesicles were derived from the plant or the fungus (Micali et al., 2011). In other studies, EVs from fungal pathogen *Paracoccidioides brasiliensis and H. capsulatum* were reported to transport antioxidants (superoxide dismutase and catalase B) and heat shock proteins (Hsp60 and Hsp70) which may have an essential role in the fungal defense mechanism (Albuquerque et al., 2008; Vallejo et al., 2012). Only recently, Hsp60 was also reported in the proteome of EVs from the fungus *Alternaria infectoria*. Several species of this genus Alternaria are also considered major plant pathogens (Silva et al., 2014).

Biochemical analyses of EVs from various human fungal pathogens has revealed the presence of a variety of lipids, proteins and RNA (Peres Da Silva et al., 2015). Although observed by microscopy, EVs have not been isolated and characterized from a plant pathogenic fungus. Nevertheless, upon uptake by the plant cell, it is possible that the contents could modulate the plant response to the invading fungal pathogen by attenuating the immune response. Similarly, plant exosomes are poorly characterized and their composition is largely unknown. However, it is plausible that some plant exosomes, particularly those produced in response to fungal threat, might contain small molecules and proteins that are toxic to the fungus.

Finally, cell wall remodeling is a key process on both sides of the plant-fungus interaction (Bellincampi et al., 2014). Proteomics studies highlighted the presence of various enzymes (Endochitinase 1 precursor, Beta-glucosidase 4, Beta-1,3-glucanosyltransferase 3, and Chitin synthase B) in EVs secreted by *H. capsulatum*. Similarly, *S. cerevisiae* secreted more than 20 proteins implicated in cell wall assembly including glucanases and glucanosyl transferases (Oliveira et al., 2010b). These enzymes have the capacity to regulate synthesis and hydrolysis of cell wall components highlighting the potential role of EVs in cell wall remodeling (Albuquerque et al., 2008). Fungal cell wall synthesis is also known to be mediated by chitosomes, small vesicles containing chitin microfibrils, in *Neurospora crassa* (Riquelme et al., 2007). Chitosomes follow an unconventional secretory pathway to transport various components of the chitin synthase family required for fungal cell wall synthesis. Whilst the differences between chitosomes and EVs are not clearly understood, it can be speculated that EVs can also play a pivotal role in fungal cell wall remodeling. In plants, reinforcement of the cell wall is one of the major strategies of the host to restrain further invasion by the pathogen (Lionetti and Métraux, 2014). The delivery of the cell wall carbohydrates to the extending chains of insoluble polysaccharides that make up majority of the cell wall is relatively poorly understood. The role of EVs in cell wall remodeling in both the fungus and plant is understudied and needs further research.

## Conclusion

**Figure 1:**
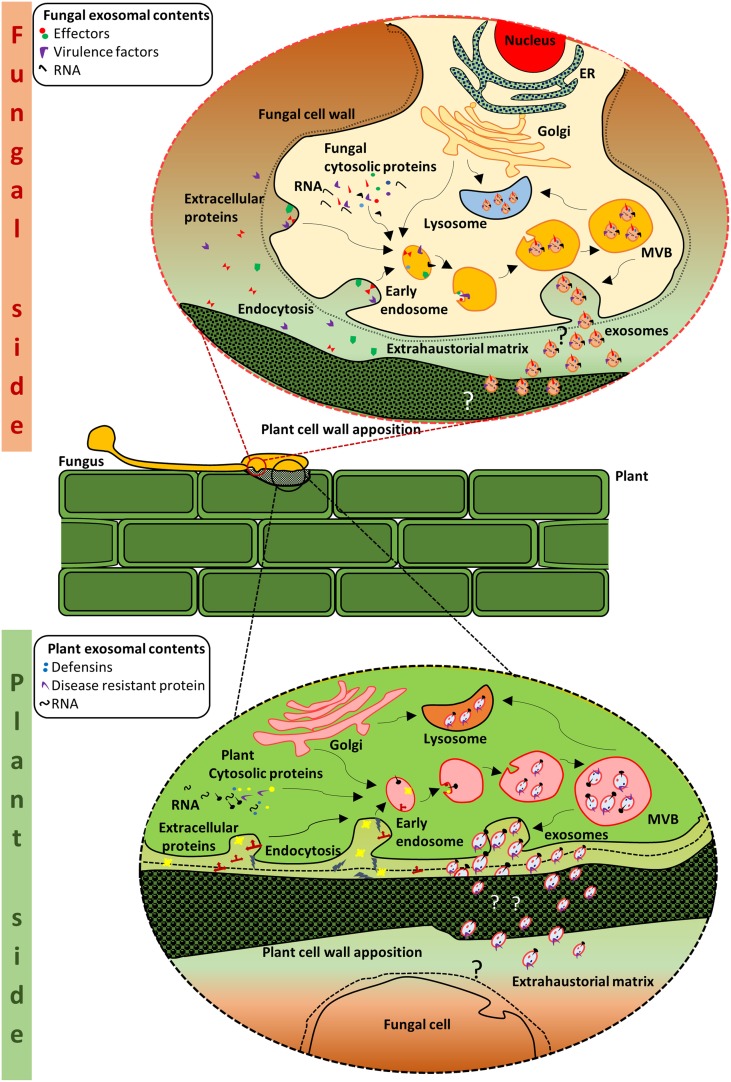
**Schematic representation of putative crosstalk via EVs at the plant-fungal interface**. Exosome biogenesis and secretion in the fungal side: MVBs are formed from the early endosomes. Within the MVBs, invagination of the limiting membrane results in the formation of intraluminal vesicles which are packaged with protein and RNA cargo from the cell. The MVBs either fuse with the plasma membrane or with the lysosome for degradation. When the MVBs fuse with the plasma membrane, the intraluminal vesicles are released as exosomes. The exosomes are considered to contain various molecules including effectors that are required for the establishment of the pathogen and/or infection. Exosome biogenesis and secretion in the plant side: Similarly, on the plant side, vesicles from the MVBs may contain innate immunity proteins and defense molecules that can impede fungal growth or lead to alterations in the fungal cell wall. Thus, the plant and its fungal counterpart could utilize the exosomes as one of the many strategies in their mutual struggle for survival.

Recent findings pertaining to the role of EVs in the interaction between fungal pathogens and humans have led us to ask whether EVs also have a major role in plant pathogen interactions. It is still unknown how effectors and defense molecules are packaged and transported across the plasma membranes and cell walls of the plant and fungal cells. We propose that proteins lacking secretion signals could be packaged into EVs for passage through the plasma membrane and the cell wall (Figure 1). Alternatively, proteins containing a secretion signal could be secreted into the matrix of the cell wall and then bind to EVs via a lipid binding motif. The protein then transits the cell wall as a passenger on the outer leaflet of the vesicle. Based on the discovery that EVs aid disease progression (Boukouris and Mathivanan, 2015; Gangoda et al., 2015), we propose that EVs can mediate/aid in fungal infection. This could be achieved via the transfer of effectors via EVs and/or by modulating the host cells response in favor of the fungal pathogen. Similarly, we propose that plant EVs can aid in the protection against pathogenic infections. Upon infection, defense molecules can be packaged and delivered to the site of infection to protect against the invading pathogen. Further to this, we propose that the molecular cargo present in EVs is specific to the type of insult or infection. For instance, molecular cargo present in EVs of plants during stress can be significantly different to that produced during fungal infection. Thus the molecular cargo contained within EVs of plant or fungus can serve as indicators of health, stress, and disease. Investigation of the role of EVs in plant-fungal interactions is likely to uncover a new mechanism for delivery and identification of molecules required for a productive infection and/or defense response. This knowledge will enhance our ability to protect agricultural crops against the damaging effects of fungal pathogens and securing our food sources for generations to come.

### Conflict of interest statement

The authors declare that the research was conducted in the absence of any commercial or financial relationships that could be construed as a potential conflict of interest.
